# Assessing cognitive insight in nonpsychiatric individuals and outpatients with schizophrenia in Taiwan: an investigation using the Beck Cognitive Insight Scale

**DOI:** 10.1186/1471-244X-11-170

**Published:** 2011-10-21

**Authors:** Yu-Chen Kao, Tzong-Shi Wang, Chien-Wen Lu, Yia-Ping Liu

**Affiliations:** 1 Department of Psychiatry, Songshan Armed Forces General Hospital, Taipei, Taiwan; 2 Department of Psychiatry, Taipei Tzu Chi General Hospital, New Taipei City, Taiwan; 3 Department of Physiology, National Defense Medical Center, Taipei, Taiwan

**Keywords:** cognitive insight, self-reflectiveness, self-certainty, BCIS

## Abstract

**Background:**

The Beck Cognitive Insight Scale (BCIS) was designed for the assessment of the cognitive processes involved in self-reflection and the ability to modify erroneous beliefs and misinterpretations. Studies investigating the factor structure of the BCIS have indicated a two-factor model in the psychotic population. The factor structure of the BCIS, however, has not received much consideration in the nonpsychiatric population. The present study examined the factor structure and validity of the BCIS and compared its scores between nonpsychiatric individuals and outpatients with psychosis.

**Method:**

The Taiwanese version of the BCIS was administered to 507 nonpsychiatric individuals and 118 outpatients with schizophrenia. The psychometric properties of the BCIS were examined through the following analyses: exploratory and confirmatory factor analyses, reliability, correlation analyses, and discriminative validity.

**Results:**

The BCIS showed adequate internal consistency and stability over time. Exploratory and confirmatory factor analyses on the 15-item measure indicated a two-factor solution that supported the two dimensions of the Taiwanese BCIS, which was also observed with the original BCIS. Following the construct validation, we obtained a composite index (self-reflectiveness minus self-certainty) of the Taiwanese BCIS that reflected cognitive insight. Consistent with previous studies, our results indicated that psychosis is associated with low self-reflectiveness and high self-certainty, which possibly reflect lower cognitive insight. Our results also showed that better cognitive insight is related to worse depression in patients with schizophrenia spectrum disorders, but not in nonpsychiatric individuals. The receiver operating characteristic (ROC) analyses revealed that the area under the curve (AUC) was 0.731. A composite index of 3 was a good limit, with a sensitivity of 87% and a specificity of 51%.

**Conclusion:**

The BCIS proved to be useful for measuring cognitive insight in Taiwanese nonpsychiatric and psychotic populations.

## Background

Individuals who are diagnosed with schizophrenia frequently disagree with mental health professionals regarding the nature of their experiences and whether they are in need of psychiatric treatment, such as medication [1,2]. This phenomenon, which is often referred to as "lack of awareness" or "poor insight," has been linked to poor medication compliance [3,4] and clinical outcome [3-5]. A number of etiological models, such as the psychological defense [4,6,7], clinical [4,8], and neuropsychological [3,4,6,9] models, have been proposed to explain the poor clinical insight in schizophrenia.

Neuropsychological impairment has been suggested as a central factor underlining poor insight. Poor insight is associated with a secondary deficit in neurocognition due to structural [10-12] and/or functional brain deficits [4,13], especially frontal or parietal dysfunction [14,15]. Aleman et al. (2006) [9] have demonstrated that poor insight is associated with poor functioning in a range of cognitive domains, including intelligence quotient (IQ), memory, and the set-shifting and error-monitoring aspects of executive function. Several studies have also noted that metacognition has the potential to influence insight in individuals with schizophrenia [16-18]. The term "metacognition" was first coined by Semerari et al [19], and is defined as the "general capacity to think about thinking" [19,20]. Of note, metacognition is considered to concern a wide range of internally and socially driven cognitive acts [19,21]. Through metacognition, individuals not only process information that they encounter, but they can also react to and think about their own mental states and those of others [19,21]. Specifically, there is growing evidence [17,18] that metacognition is not only one of many clinical or psychological variables linked to insight in schizophrenia, but metacognition is also a factor that moderate the effects of other factors such as self-reflectivity (the ability to comprehend one's own mental state) [17-19,21,22] and mastery (the ability to form knowledge about one's own mental states and those of others and to use that knowledge to response to psychological challenges) [17-19,23] that underline an individual's awareness of illness. Lysaker et al. (2011) [18], for instance, suggested that metacognitive abilities, rated by the Metacognition Assessment Scale [19], may be linked to insight in individuals with schizophrenia independent of concurrent impairments in neurocognition. Thus, poor insight in schizophrenia may result in part from deficits in metacognitive capacities, namely self-reflectivity and mastery.

According to Beck et al. (2004), an important extension of the insight concept was introduced with the description of "cognitive insight," which was defined as a patient's current capacity to evaluate his or her anomalous experiences and atypical interpretations of events [24,25]. Those authors provided a conceptual dissociation between clinical and cognitive insight, suggesting that cognitive insight is a form of cognitive flexibility that involves first an ability to distance oneself from distorted beliefs and misinterpretations and then to reappraise these beliefs and recognize erroneous conclusions [24,25]. In a more specific way, at least four aspects of cognitive insight can be influenced by psychosis, according to Beck and Warman's research [25]: (a) impairment of the ability to be objective concerning delusional experiences and cognitive distortions, (b) reduced capacity to put these experiences into perspective, (c) unresponsiveness to corrective information from others, and (d) overconfidence in delusional judgments. Recent findings have also highlighted the potential importance of cognitive insight as a mediator of response to cognitive behavioral therapy of psychosis [26]. In fact, Beck's theory offered a stronger theoretical basis for cognitive insight and supported the contention that it is both identifiable and quantifiable [24,25]. Thus, cognitive insight was first operationalized with the publication of the 15-item Beck Cognitive Insight Scale (BCIS) [24,25].

The initial study by Beck et al. showed that the BCIS could measure individuals' capacity for distancing themselves from and re-evaluating anomalous beliefs and misinterpretations [24,25]. The BCIS, which is a 15-item self-report measure, is composed of two subscales: self-reflectiveness (SR) and self-certainty (SC) [24,25]. The former includes items measuring objectivity, reflectiveness, and openness to feedback, and the latter measures the certainty of one's beliefs and judgments [24,25]. Beck and colleagues proposed that high levels of certainty might diminish the capacity for self-reflection; thus, a composite index providing an estimate of overall cognitive insight was calculated by subtracting the score for the SC subscale from the score for the SR subscale [24,25]. The two subscale scores were only weakly intercorrelated, which indicated that they represent two different dimensions of cognitive insight [24]. The BCIS in this regard proved to be an indirect tool for evaluating the impairment of the 'higher level' functions in schizophrenia, and, more specifically, impairment in the process of distancing oneself from highly salient (delusional) beliefs and viewing them in terms of executive functions [24,25,27].

The majority of studies that have investigated the relationship between the overall cognitive insight of schizophrenia patients (measured by the composite index scale of the BCIS) and clinical insight (measured by the Scale to Assess Unawareness of Mental Disorder [24,28-30], the Positive and Negative Syndrome Scale [29-33], and the Birchwood Insight Scale [34]) have found that these two variables are significantly related and demonstrate convergent and criterion validity, respectively. The reliability and validity of the BCIS have been demonstrated in a mixed group of inpatients with psychosis and depression [24,35], groups of inpatients or outpatients with schizophrenia spectrum disorders [28,31,34], and a group of patients with bipolar disorder [32,35]. The BCIS has also been applied to nonclinical populations [32,33,36-38] and the internal consistency of the BCIS is similar between clinical and nonclinical samples [32,37].

A review of the literature indicates that clinical insight is associated with depression in patients with psychosis [33,39-41]; however, the findings of studies examining the relationship between cognitive insight and depression have been mixed. Two studies [33,37] found a correlation between depression as measured by the Beck Depression Inventory-II (BDI-II) and cognitive insight in patients with schizophrenia or schizoaffective disorder; however, another study [24] did not find such a correlation in individuals with psychotic disorders. In addition, Pedrelli et al. did not find an association between cognitive insight and depression as measured by the Hamilton Rating Scale for Depression in middle-aged and older patients with schizophrenia and schizoaffective disorder [34]. However, in an investigation of psychotherapy for individuals with schizophrenia or schizoaffective disorder, Granholm et al. (2005) [26] discovered a relationship between increased cognitive insight and increased depression midway through the treatment. To date, researchers have become increasingly interested in the relationship between cognitive insight and depression in individuals with psychotic disorders. However, there has thus far been relatively little research into this area using a non-psychiatric population sample. Two studies that investigated the BCIS with a normal population did not include a measure of depression [36,38], which makes it difficult to draw general conclusions about the relationship between cognitive insight and depression.

The BCIS has been used for comparisons between individuals with a psychotic diagnosis and healthy controls. One earlier study reported that patients scored significantly higher than controls on SC, but differences in SR were not observed between the two groups [37]. In another study, Engh et al. (2007) [32] found no difference in SR or SC subscales between individuals with schizophrenia, those with bipolar disorder, and normal controls. However, Martin et al. (2010) [38] found that healthy controls exhibited higher SR, lower SC, and a higher composite index than patients with schizophrenia. The failure of some previous studies to differentiate the SR between patients and controls could be due to a high percentage of SR items being omitted by the controls [32,38], cultural differences in the way individuals understand questions on the scale [37,38], and insufficient sample size [32,37,38]. At present, high scores on the SR subscale and low scores on the SC subscale are regarded as being normal [24,25,38,42]. However, this theoretical view has not been sufficiently supported by direct research to clarify possible impairments in psychosis and to answer the question as to whether increased SR and decreased SC are evidence of improvement [38]. In addition, cutoff scores that would allow a categorical determination of the presence or absence of impaired cognitive insight, as measured by the composite index, have not been clearly determined. In light of these concerns, the present study investigated the psychometric properties and factor structure of the BCIS with a large nonpsychiatric population and compared the results to those collected from individuals with schizophrenia.

Researchers and clinicians have expanded upon Beck's original work with numerous studies focusing on the extent to which the construct of the BCIS contributes to our understanding of a wider range of cognitive insight. To date, the BCIS has already been translated into several languages, including Turkish [28], French [31], Norwegian [32], Japanese [33], Spanish [43], Korean [44], Chinese [45], and Taiwanese [46], and its validity and psychometric properties have been reported in each of these languages. Research focusing on the discriminative properties of the BCIS (i.e., thresholds that draw on the combination of sensitivity and specificity) is scarce, however. To date, only one study has reported that the composite index reliably discriminates between outpatients and nonpsychiatric individuals (i.e., AUC, 0.641; SE, 0.033; 95% Confidence interval (95% CI), 0.575-0.707; nonparametric P < 0.001) [38]. Visual inspection of the ROC curve suggested that no single part of the curve maximizes specificity and sensitivity [38].

Although a growing number of researchers have considered the potential of using self-report scales in cognitive insight assessments, very little attention has been given to the influence of cultural background on Taiwanese individuals' particular beliefs regarding cognitive insight. The extent to which the two-factor model of Beck et al. [24,25] can be generalized to our nonpsychiatric population is unclear. Therefore, the aim of the present study was threefold. The first purpose was to provide reference data for the BCIS, i.e., examine its reliability and validity in a large sample of individuals with or without psychiatric diagnoses in Taiwan. We predicted that the factor structure of the Taiwanese version of the BCIS would be similar to the findings of the studies by Beck et al. (2004) [24,25] and Pedrelli et al. (2004) [34]. Based on the factor analyses of the BCIS that we performed on data from a nonpsychiatric population, we make recommendations as to how the BCIS should be scored and interpreted, and we also explore the ability of the BCIS to discriminate between participants with and without psychosis. In view of the results from earlier studies [33,37,38], we hypothesized that individuals with schizophrenia would have less total cognitive insight than nonpsychiatric individuals. To address the previously outlined issues and to begin to fill in the gaps of previous research, the second purpose of the present study was to investigate the role of depression in cognitive insight, looking at nonpsychiatric and psychotic populations. The final purpose of the present study was to present additional statistical support for the BCIS, using a ROC curve analysis [47].

## Method

### Participants

This study was performed in accordance with the latest version of the Declaration of Helsinki. Prior to commencing this study, its performance approval was obtained from the local Research Ethics Committee. Following a comprehensive explanation of this study, informed consent was obtained from all of participants. Participation in the present study was strictly voluntary and anonymous.

Two groups of participants were studied. A total of 130 Taiwanese outpatients (58 males, 72 females) were recruited from one psychiatric outpatient department of a general hospital located in Taipei, a city in the North of Taiwan. Outpatients were diagnosed based on a Structured Clinical Interview for Diagnostic and Statistical Manual of Mental Disorders (Fourth Edition) [48] for at least two years and verified by evaluations that were conducted by at least two independent evaluators after at least six months of continuous observation. Patients who showed evidence of mental retardation (i.e., a Mini-Mental State Examination score < 23, which indicated disorientation or cognitive impairment to an extent that could interfere with the extensive clinical assessment [49]) or organic brain pathology, including cerebral tumor, epilepsy, systemic disease, history of cranial trauma, brain surgery, or history of substance abuse or dependence in the past or present, were excluded from the study. Of the 130 patients initially invited to participate in the study, twelve (4 males, 8 females) patients did not complete the cognitive insight questionnaire, which left a pool of 118 participants (54 males, 64 females) who were available for analyses (91% of the initial sample). Of the 118 patients, 76 were diagnosed with schizophrenia and 42 with schizoaffective disorder. The mean age and formal education of the patients were 39.27 years (SD, 9.86; range, 20-59) and 12.64 years (SD, 2.52; range, 9-18), respectively. The mean age of illness onset was 24.39 years (SD, 7.06; range, 15-43), the mean illness duration was 15.02 years (SD, 9.51; range, 3-37), and the patients had an average of 7.11 lifetime psychiatric hospitalizations (SD, 4.73; range, 2-25). Prior to entering the study, all patients received atypical antipsychotic medications.

We preferred nonpsychiatric participants with the same-gender who lived in the same residential area as each patient to avoid the sociocultural bias: 38 individuals refused to participate in the study, which yielded a total final sample of 507 nonpsychiatric controls (93% of the initial sample) to serve as a general population comparison group (231 males and 276 females; age range 18-65 years; mean age, 35.08 years; SD, 10.87). The mean formal education for individuals in the control group was 15.21 years (SD, 2.17; range, 9-22).Only participants who denied having received a formal diagnosis of a mental illness and/or a formal specific treatment for a mental illness and did not have a history of either neurologic impairment or substance abuse were selected.

Baseline demographic data consisted of gender, age, marital status (e.g., unmarried, married, divorced), religious beliefs (e.g., nonreligious, Buddhism, Taoism, Christianity, Catholicism) and formal educational attainment. A semistructured interview to determine the age of illness onset, the duration of illness, and recurrence of lifetime psychiatric hospitalizations was obtained from the responsible psychiatrist. Data were also extracted from all available information, including hospital records and information from family members. The age of illness onset was defined as the age when the patient met DSM-IV criteria [48] for the first time. The duration of the illness was defined as the time since the first psychotic episode. Sociodemographic characteristics for all participants are presented in Table 1. The repeated measures Chi-square and Student's t tests showed that the two groups differed significantly in age (t = 3.84), educational attainment (t = -10.27), marital status (χ^2 ^= 61.36), and religious beliefs (χ^2 ^= 29.36) were detected (P < 0.01 for all variables).

**Table 1:** Sociodemographic and clinical characteristics of participants (n = 625)

	Nonpsychiatric group (n = 507)		Psychotic group (n = 118)	
**Variables**	**n**	%	**n**	%

**Gender**				
Male	231	45.4	54	45.7
Female	276	54.6	64	54.3
**Age**				
18-29	172	33.9	19	16.1
30-39	203	40.0	48	40.7
40-49	71	14.0	24	20.3
50-59	42	8.4	27	22.9
60-69	19	3.7	0	0
**Marital status**				
Unmarried/single	271	53.4	106	89.8
Married	221	43.6	6	5.1
Divorced	15	3.0	6	5.1
**Religion**				
None	265	52.3	47	39.8
Buddhism	148	29.2	58	49.2
Taoism	65	12.8	5	4.2
Christianity	29	5.7	5	4.2
Catholicism	0	0	3	2.5

	**Mean** **(SD)**	**Range**	**Mean** **(SD)**	**Range**

**Age (yr)**	35.08 (10.87)	18-65	39.27 (9.86)	20-59
**Education (yr)**	15.21 (2.17)	9-22	12.64 (2.52)	9-18
**Average BDI-II** **scores**	4.83 (7.13)	0-36	19.69 (14.97)	2-57

To identify the test-retest reliability of the Taiwanese BCIS measure in this study, 100 participants, including 50 from each diagnostic group, completed the Taiwanese BCIS again four weeks after the initial assessment. All 50 outpatients were closely followed up with the same investigator during the time between assessments, which permitted a longer interval to complete the test-retest procedure.

### Measurements

#### Taiwanese version of the Beck Cognitive Insight Scale

The BCIS [24,25] is a standardized self-rated instrument that is composed of 15 items that measure cognitive insight. Kao et al. (2010) [46] previously administrated a Taiwanese validated version of the BCIS, which was translated into Taiwanese and back translated into English for semantic congruence. The Taiwanese BCIS consists of two subscales, reflective attitude (9 items) and certain attitude (6 items) [46], which are different from the original BCIS. Evidence of initial reliability and validity has been reported elsewhere [46].

#### Beck Depression Inventory II

Because the impact of depression on cognitive insight in nonclinical and clinical samples is unknown, measures assessing depression were included to control for a potential between-group effect. Thus, the Beck Depression Inventory II (BDI-II) was administered. This scale consists of a 21-item self-report scale [50], and each item consists of four alternative statements that reflect gradations in the intensity of a particular depressive symptom (rated in terms of severity from 0 to 3). The resulting scores are summed to obtain a total depression score (range, 0-63). The BDI-II showed generally acceptable internal consistencies in the present study (alpha, 0.95 for the nonpsychiatric group and 0.90 for the psychotic group).

### Statistical analysis

All statistical tests were performed using the Statistical Package for the Social Sciences (SPSS) version 15.0 and Analysis of Moment Structures (AMOS) version 19.0 for Windows (SPSS Inc., Chicago, IL, USA). P values of 0.05 or less were taken to indicate the statistical significance of the two-tailed test results.

After the administration of the Taiwanese BCIS to the nonpsychiatric (n = 507) controls, we used the exploratory factor analysis (EFA) method to extract factors. The number of factors was determined by an examination of scree plots and the size of eigenvalues. An orthogonal (varimax) rotation was made to achieve a more readily interpretable factor structure. We extracted factors with eigenvalues greater than or equal to 1.0 during the exploratory phase of the study. In addition, we chose 0.4 as a cutoff for the size of loading to be interpreted [51]. Correction analyses and the test-retest procedure for temporal stability assessment were performed using Pearson product-moment correlation coefficients. Internal consistency of each subscale resulting from the EFA was determined with Cronbach's alpha values, and the accepted level was set at 0.7 [51].

Confirmatory factor analyses (CFA) were conducted to test the hypothesized factorial structure of the Taiwanese BCIS. The sample size and the number of participants for each observable variables were sufficient for conducting CFA, and we followed the recommendations of Anderson and Gerbing (1984) [52], Bentler and Chou (1987) [53], and Garson (2007) [54]. Following the recommendations of Beck et al. (2004) [24,25], Pedrelli et al. (2004) [34], Uchida et al. (2009) [33], and Martin et al. (2010) [38], the items for the cognitive insight scale were divided into two "parcels" to produce more robust estimates. Therefore, four models with 15 items each were specified in the present study. The first model (Model 1) was a one-factor model, which suggested a single cognitive insight factor for grouping all 15 items. The second model (Model 2) was based on the results of the EFA in the present study. The third model (Model 3) was the original two-factor model proposed by Beck et al. (2004) [24,25], Pedrelli et al. (2004) [34], Uchida et al. (2009) [33], and Martin et al. (2010) [38], which included a nine-item subscale representing self-reflectiveness and a six-item subscale representing self-certainty. The fourth model (Model 4) was a two-factor model that was recently hypothesized by Kao et al. (2010) [46]. Nine items represented a reflective attitude factor and six items represented a certainty factor. AMOS 19 was used to perform the CFA for each of the four models in the nonpsychiatric (n = 507) and psychotic (n = 118) groups separately. In the present study, model fit was evaluated based on several goodness of fit indices, including the goodness of fit index (GFI), adjusted GFI (AGFI), non-normed fit index (NNFI), comparative fit index (CFI), Tucker-Lewis index (TLI), root mean square error of approximation (RMSEA), and the Chi-square statistic. Because the Chi-square test is sensitive to sample size, other fit indices were considered [55,56]. An adequate fit of the model to the data is generally indicated by values of > 0.9 for GFI, AGFI, NNFI, CFI, and TLI; values of < 0.08 for RMSEA; and nonsignificant chi-square statistics (P > 0.05), which indicate a lack of differences between the predicted and actual models [55,56].

Correlation analyses were conducted to determine the relationship between the BCIS and the BDI-II scores in the two groups. Student's t tests and one-way analyses of variance (ANOVA), when appropriate, were used to determine whether clinical variables were significantly different between the subgroups of participants. Student's t tests were performed to ensure that the BCIS subscales and index scores would differentiate between psychotic outpatients (n = 118) and nonpsychiatric controls (n = 507). To evaluate the impact of the clinical variables on cognitive insight independent of sociodemographic variables, we conducted analyses of covariance (ANCOVA) in which the BCIS subscales and index scores were dependent variables, the groups were independent variable, and age, education, marital status, and religion were covariates (concomitant variables). In addition, we also conducted an ANCOVA to test whether cognitive insight was associated with psychiatric diagnoses independent of depressive symptom severity (i.e., the subscales and index scores were dependent variables, group were the independent variables, and depression was the covariate).

To explore the discriminatory power of the Taiwanese BCIS, ROC analyses were utilized to evaluate overall performance (i.e., the area under the curve) and the performance at the optimal thresholds (sensitivity and specificity) of the Taiwanese BCIS scale. Furthermore, Youden's index [57], which assesses the maximum potential effectiveness of a test, was calculated to determine which cutoff points on the BCIS maximized both sensitivity and specificity. Youden's index is calculated by subtracting one from the sum of a test's sensitivity and specificity (i.e., the value is expressed as a part of a whole number rather than a percentage and maxium (sensitivity + specificity) - 1) [58,59]. The area under the curve (AUC) of the ROC represents the diagnostic efficiency of a given measurement based on the method developed by Hanley and McNeil (1982) [60]. Because this study was designed to provide practical thresholds that could serve as clinical markers with acceptable discriminability, we focused on the thresholds that were obtained when the AUC was 0.7 or above [61]. ROC analyses were performed using MedCalc for Windows, Version 9.2.1.0 (MedCalc Software, Mariakerke, Belgium).

## Results

### Construct validity and reliability of the Taiwanese BCIS

Prior to the EFA, the Kaiser-Meyer-Olkin measure of sampling adequacy was at an acceptable level of 0.75, and Bartlett's test of sphericity was significant (1485.06, P < 0.001), which indicated the adequacy of the data for applying the EFA. According to the EFA with varimax rotation, the first two eigenvalues were 3.14 and 2.37, which accounted for 39.5% of the total variance. These eigenvalues indicated that two factors should be extracted and inspected for simple structure.

Each of the subscales was developed based on the factor loadings and applied in the subsequent analyses. For each item, the highest factor loading determined the subscale inclusion. The two subscales that were indicated by the analyses can most suitably be described as the SR subscale and the SC subscale (Table 2). Based on concepts regarding self-correction that were derived from previous studies [21,22,30,31,35], a composite index was calculated (i.e., SR minus SC) as the measure of cognitive insight in this study.

**Table 2:** Factor analysis and reliability of the Taiwanese BCIS

			BCIS^a)^		BCIS^b)^	
			**(n = 507)**		**(n = 180)**	
			**Factor**		**Factor**	

**Item**	**Statement**	**Subscale**	**I**	**II**	**I**	**II**

1	At times, I have misunderstood other's attitudes toward me.	SR	0.48	0.11	0.50	0.06
2	My interpretations of my experiences are definitely right.	SC	0.25	0.48	0.08	0.69
3	Other people can understand the cause of my unusual experiences better than I can.	SR	0.55	-0.10	0.60	0.10
4	I have jumped to conclusions too fast.	SR	0.60	0.09	0.61	0.09
5	Some of my experiences that have seemed very real may have been due to my imagination.	SR	0.62	0.24	0.76	-0.01
6	Some of the ideas I was certain were true turned out to be false.	SR	0.58	0.14	0.62	-0.14
7	If something feels right, it means that it is right.	SC	0.16	0.52	0.12	0.62
8	Even though I feel strongly that I am right, I could be wrong.	SR	0.42	-0.24	0.31	0.25
9	I know better than anyone else what my problems are	SC	0.03	0.65	0.05	0.79
10	When people disagree with me, they are generally wrong.	SC	0.02	0.59	0.49	0.20
11	I cannot trust other people's opinion about my experiences.	SC	-0.13	0.66	0.55	0.03
12	If somebody points out that my beliefs are wrong, I am willing to consider it.	SR	0.50	-0.22	-0.01	0.47
13	I can trust my own judgments at all times.	SC	0.07	0.69	0.07	0.69
14	There is often more than one possible explanation for why people act the way they do.	SR	0.54	-0.25	0.04	0.55
15	My unusual experiences may be due to my being extremely upset or stressed.	SR	0.46	0.07	0.34	0.18
	% of Variance		22.0	17.5	28.3	17.7
	Cronbach's alpha coefficient		0.75	0.78	0.7	0.72

Note: Extraction with Rotation method: principal component analysis with Varimax

SR indicated Self-reflectiveness subscale; SC, Self-certainty subscale.

a): Translated Taiwanese version of the Beck Cognitive Insight Scale, administered to Taiwanese 508 nonpsychiatric controls.

b): Translated Taiwanese version of the Beck Cognitive Insight Scale, administered to native Taiwanese 60 outpatients with schizophrenia, 60 outpatients with major depressive disorders, and 60 nonpsychiatric controls.

The results of CFA for the four models of the factor structure of the BCIS in the Taiwanese populations are shown in Table 3. Model 2 and Model 3 adequately fulfilled the criteria for good fit in the nonpsychiatric and psychotic groups, whether the other factorial models (i.e. GFI, AGFI, NNFI, CFI, and TLI) had values below the recommended threshold of 0.9 or RMSEA had a value above 0.08. We therefore concluded that the CFA demonstrated superiority of the original two-factor model, and proceeded to assess the internal consistency reliability of subscales calculated from sum scores created by summing the items loading on a given factor.

**Table 3:** Confirmatory factor analysis for four models of factor structure of the Taiwanese BCIS in two groups (n = 625)

	Fit indices						
	
Models	χ^2^ (df)	GFI	AGFI	NNFI	CFI	TLI	RMSEA (90%CI)
**Factor structure for the nonpsychiatric group (n = 507)**							
**Model 1**	371.84 (90)	0.72	0.69	0.71	0.69	0.69	0.14 (0.136-0.144)
**Model 2**	296.57 (89)	0.94	0.91	0.89	0.92	0.91	0.058 (0.052-0.064)
**Model 3**	296.57 (89)	0.94	0.91	0.89	0.92	0.91	0.058 (0.052-0.064)
**Model 4**	335.57 (89)	0.83	0.80	0.79	0.81	0.80	0.091 (0.083-0.099)
**Factor structure for the psychotic group (n = 118)**							
**Model 1**	241.42 (90)	0.69	0.61	0.67	0.68	0.66	0.158 (0.152-0.164)
**Model 2**	162.07 (89)	0.91	0.90	0.88	0.89	0.90	0.071 (0.062-0.08)
**Model 3**	162.07 (89)	0.91	0.90	0.88	0.89	0.90	0.071 (0.062-0.08)
**Model 4**	213.13 (89)	0.80	0.73	0.78	0.81	0.77	0.099 (0.092-0.106)
**Criteria^a^**	**n.s.**	**> 0.9**	**> 0.9**	**> 0.9**	**> 0.9**	**> 0.9**	** < 0.08**


*P < 0.05; **P < 0.01

Note: Model 1 indicated one-factor (unidimensional); Model 2, two-factor based on the results of EFA in this study; Model 3, two-factor based on the original BCIS studies; Model 4, two-factor based on the Kao et al. study (2010).

Abbreviations: GFI, Goodness of Fit Index; AGFI, Adjusted Goodness of Fit Index; NNFI, Non-normed Fit Index; CFI, Comparative Fit Index; TLI, Tucker-Lewis index; RMSEA 90% CI, Root Mean Square Error of Approximation 90% confidence interval; n.s., non-significant (P > 0.05).

^a ^Adapted from Petersens (2009); Hoyle and Panter (1993)

Internal consistency analyses were conducted on each of the two subscales, and the reliabilities (coefficient alpha) of the two subscales of the Taiwanese BCIS for the 507 controls were 0.75 for the SR and 0.78 for the SC. In addition, the alpha coefficients for the SR and SC were 0.72 and 0.68, respectively, for the 118 outpatients with schizophrenia or schizoaffective disorder. The test-retest reliability coefficient over a four-week interval ranged from 0.75 to 0.78 for the subscales and composite index level in the two groups (P < 0.01 for all values). It should be noted, however, that no significant correlation was found between the SR and SC scores in this study.

### Psychotic disorder and nonpsychiatric control comparisons

Table 4 presents the demographic and clinical characteristics of the two groups as well as their scores on the BCIS according to sociodemographic variables. In the nonpsychiatric group, there were no significant differences among the BCIS scores with respect to religion. Interestingly, women scored higher on both the SR and SC compared with men (t = 2.04, P = 0.04; t = 3.7, P < 0.01, respectively). Moreover, the SC scores were different with regard to age (F _(4, 502) _= 5.81, P < 0.01) and marital status (F _(2, 504) _= 5.86, P < 0.01). Among participants with psychosis, the composite index scores differed significantly in terms of age (F _(3, 114) _= 3.78, P = 0.012). Student's t tests and ANOVA results also indicated that there were no significant differences in the subscales and index scores between sex, marital status, or religion (all P > 0.05 in all comparisons) in the psychotic outpatients.

**Table 4:** Distribution of scores on the Taiwanese BCIS among the samples (n = 625)

	Nonpsychiatric group (n = 507)			Psychotic group (n = 118)		
	**SR** **Mean** **(SD)**	**SC** **Mean** **(SD)**	**Composite index** **Mean** **(SD)**	**SR** **Mean** **(SD)**	**SC** **Mean** **(SD)**	**Composite index** **Mean** **(SD)**

**Gender**						
Male	14.41 (3.42)	8.42 (2.41)	5.99 (3.59)	11.93 (4.91)	8.98 (3.83)	2.94 (4.61)
Female	13.82 (3.10)	7.60 (2.59)	6.21 (3.45)	12.39 (3.93)	9.25 (3.22)	3.14 (3.43)
t	2.04	3.7	-0.72	0.56	0.41	0.26
P	0.04*	< 0.01**	> 0.05	> 0.05	> 0.05	> 0.05
**Age**						
18-29	14.16 (2.95)	7.54 (2.35)	6.62 (3.38)	11.26 (3.84)	8.16 (2.61)	4.11 (2.64)
30-39	13.68 (3.13)	7.80 (2.70)	5.88 (3.33)	11.38 (4.82)	9.60 (4.05)	1.77 (3.45)
40-49	14.63 (3.44)	8.56 (2.38)	6.07 (3.87)	13.04 (3.41)	9.46 (2.98)	3.58 (3.81)
50-59	14.45 (3.74)	8.80 (1.95)	5.64 (4.06)	13.48 (4.48)	8.67 (3.40)	4.81 (5.11)
60-69	14.84 (4.95)	9.63 (2.79)	5.21 (3.77)	0 0	0 0	0 0
F	1.69	5.81	1.63	1.96	1.01	3.78
P	> 0.05	< 0.01**	> 0.05	> 0.05	> 0.05	0.012*
**Marital status**						
Unmarried/single	14.06 (3.05)	7.61 (2.56)	6.45 (3.29)	12.27 (4.44)	9.12 (3.58)	3.08 (4.03)
Married	14.09 (3.54)	8.39 (2.46)	5.70 (3.66)	12.0 (1.67)	9.50 (2.74)	2.50 (1.97)
Divorced	14.53 (2.77)	9.27 (2.43)	6.27 (4.71)	10.67 (5.61)	7.50 (2.59)	3.17 (5.19)
F	0.147	5.86	2.80	0.381	0.701	0.061
P	> 0.05	< 0.01 **	0.062	> 0.05	> 0.05	> 0.05
**Religion**						
None	14.24 (3.29)	7.98 (2.71)	6.26 (3.40)	12.32 (4.49)	8.78 (3.43)	2.53 (4.46)
Buddhism	13.89 (3.36)	7.99 (2.35)	5.90 (3.76)	11.88 (4.66)	9.29 (3.70)	2.59 (3.77)
Taoism	13.97 (3.10)	7.90 (2.20)	6.06 (3.34)	14.6 (3.21)	11.0 (2.65)	3.60 (2.88)
Christianity	13.93 (2.90)	7.93 (2.63)	6.00 (3.71)	11.8 (1.30)	8.20 (3.63)	3.60 (3.91)
Catholicism	0 0	0 0	0 0	12.33 (2.08)	9.67 (1.15)	2.67 (3.21)
F	0.424	0.021	0.352	0.461	0.599	0.413
P	> 0.05	> 0.05	> 0.05	> 0.05	> 0.05	> 0.05

*P < 0.05; **P < 0.01

To assess the discriminative validity of the Taiwanese BCIS, we used Student's t tests to compare the mean test scores of the subscales and the composite index for the patients and controls. The results are presented in Table 5. Before discussing the potential confounding effects of clinical variables with ANCOVA, certain facts that were manifested in the patients and controls are worth considering. For examples, there was a statistically significant difference in the BCIS scores between the psychotic and healthy control samples. The mean composite index scores were significantly higher in the 507 nonpsychiatric controls than in the outpatients with psychosis (t = -8.32; P < 0.01). With regard to the scores for the two subscales, the SR scores of the outpatients were lower than the scores of the controls (t = -4.44, P < 0.01). The SC scores of the outpatients, however, were higher than the scores of the controls (t = 3.38, P < 0.01). Adjusting for sociodemographic variables such as age, education, marital status, and religion in the ANCOVAs did not have any significant effects on these results.

**Table 5:** Descriptive statistics for nonpsychiatric control and psychotic outpatient groups and the results of an ANCOVA with depressive symptoms scores as a covariate showing differences between the two groups

	Controls (n = 507)		Outpatients (n = 118)			
	**Mean** **(SD)**	**Range**	**Mean** **(SD)**	**Range**	**t-test**	**ANCOVA** **F(1,623)**

**SR**	14.09 (3.26)	4-24	12.18 (4.39)	1-25	-4.44**	20.72**
**SC**	7.97 (2.49)	0-15	9.13 (3.50)	1-18	3.38**	11.29**
**Composite index**	6.11 (3.50)	-5-18	3.05 (3.99)	-5-18	-8.32**	48.39**

**P < 0.01

To determine whether depression was related to BCIS scores in the two groups, we conducted a series of correlation analyses. The results indicated that the SR and composite index scores were significantly correlated with depression in the psychotic group (r = 0.378, P < 0.01; r = 0.32, P < 0.05, respectively). No other significant effects were observed. For the BDI-II scores, the difference between nonpsychiatric (mean = 4.83, SD = 7.13) and psychotic (mean = 19.69, SD = 14.97) groups was significant (t = 10.51, P < 0.001). Thus, for the following analyses, the BDI-II score will be entered as a covariate when the dependent variables are examined. After controlling for depressive symptoms, significant differences remained in the two groups with regard to the SR (F _(1, 623) _= 20.72, P < 0.001), SC (F _(1, 623) _= 11.29, P < 0.001), and composite index (F _(1, 623) _= 48.39, P < 0.001) (Table 5).

### ROC curve and the AUC

Diagnostic validity based on the areas under the ROC curves and the optimal cutoff points for sensitivity and specificity were used to assess the diagnostic validity between the psychotic group and the control groups, and these data are summarized in Table 6. Based on the ROC analyses, the composite index score was able to correctly classify participants into the psychosis and healthy control groups. The cutoff threshold that discriminated between patients and controls was 3, the sensitivity was 0.87, and the specificity was 0.51 (AUC, 0.731, 95% CI, 0.694-0.765, P < 0.001).

**Table 6:** Sensitivity and specificity at various cutoff points of the Taiwanese BCIS composite index for cognitive insight

Score threshold	Sensitivity (%)	Specificity (%)	Yuden Index	n (%) of patients identified at or below cutoff
Schizophrenia (n = 118)				
19	0	100	1.00	118 (100%)
18	0	100	1.00	118 (100%)
17	0.39	99.75	1.00	117 (99.2%)
16	0.99	99.15	1.00	117 (99.2%)
15	1.78	99.15	1.01	117 (99.2%)
14	2.17	98.31	1.00	117 (99.2%)
13	4.73	98.31	1.03	116 (98.3%)
12	8.48	98.31	1.07	116 (98.3%)
11	10.85	97.46	1.08	116 (98.3%)
10	16.57	92.37	1.09	115 (97.5%)
9	22.88	88.98	1.12	109 (92.45)
8	30.97	85.59	1.17	105 (89.0%)
7	40.43	82.2	1.23	101 (85.6%)
6	54.44	77.12	1.32	97 (82.2%)
5	67.26	66.10	1.33	91 (77.1%)
4	77.51	59.32	1.37	78 (66.1%)
3*	**87.18**	**50.85**	**1.38**	**70 (59.3%)**
2	92.11	37.29	1.29	60 (50.8%)
1	95.86	29.66	1.26	44 (37.3%)
0	98.42	17.80	1.16	35 (29.7%)
-1	99.0	10.17	1.09	21 (17.8%)
-2	99.41	5.93	1.05	12 (10.2%)
-3	99.8	1.69	1.01	7 (5.9%)
-4	99.8	0.85	1.01	2 (1.7%)
-5	99.9	0.65	1.01	1 (0.9%)
-6	100	0	1.00	0 (0%)

*Optimal cutoff point (maximum sensitivity and specificity) shown in bold.

## Discussion

We analyzed the responses to the Taiwanese BCIS of 507 nonpsychiatric and 118 psychotic participants to investigate whether the existing factor structures fit the data. We also examined the psychometric characteristics of the Taiwanese BCIS. Our results broadly supported the majority of the findings in literature, which have indicated that the multidomain structure of the Taiwanese BCIS is robust, the two-factor model is the optimal representation of the relationship between the items measuring cognitive insight, and the psychometric characteristics of the Taiwanese BCIS are adequate for both nonpsychiatric and psychotic populations. Furthermore, this study provided the first evidence of cross-cultural validity of the cognitive insight construct in a Taiwanese-speaking context and it supported the use of the BCIS in cross-cultural research.

Two factors, SR and SC, emerged in exploratory factor analyses using principal axis factoring and varimax rotation; the two factors accounted for 39.5% of the variance. Interestingly, the two factors were the same as the factors determined by Beck et al. in the original BCIS [24] in a sample of inpatients with schizophrenia, schizoaffective disorder, or mood disorder; however, the factors differed from a study by Kao et al. (2010) [46], conducted in samples of patients with schizophrenia, schizoaffective disorder, major depressive disorder without psychotic features, and healthy controls. The present findings were also consistent with a previous study by Martin et al. (2010) [38], which confirmed that the basic factor structure and internal consistency of the BCIS were similar for the normal population. Recently, Uchida et al. (2009) [33] also observed acceptable internal consistency of the BCIS in a sample of nonpsychiatric Japanese individuals. These findings support the generalizability of the two-factor model of cognitive insight to both nonpsychiatric and psychotic populations. The test-retest reliability intraclass coefficients of the Taiwanese BCIS confirmed the stability of cognitive insight in nonpsychiatric and psychotic populations, thus indicating the reliability of the Taiwanese BCIS.

The alpha coefficient values for the SR and SC subscale scores in the nonpsychiatric group were 0.75 and 0.78, respectively, and these values indicated that the internal consistencies of the Taiwanese BCIS subscales were adequate for research purposes. Moreover, the present sample's alpha coefficients for outpatients who had been stabilized in an outpatient setting were higher than the alpha coefficients (0.68 for SR and 0.60 for SC) that Beck et al. [24] found for their acute sample. These latter alpha coefficients, however, are similar in magnitude to those reported by Pedrelli et al. (2004) [34] (i.e., 0.7 for SR and 0.55 for SC) for 164 middle-aged and older outpatients diagnosed with either schizophrenia or a schizoaffective disorder. A partial explanation for the inconsistent results may lie in the fact that these low coefficients alpha are partially attributed to the severity of the patients' current symptoms, especially for those patients with thought disturbances and concentration difficulties [24]. In the psychotic group, the alpha coefficient for the SC scores was < 0.7, but this value was considered acceptable for the present research purpose because these subscales were composed of fewer that ten items [24,62,63].

To our knowledge, this is the largest population-based examination of the factor structure of the BCIS. Testing for multiple a priori models and indices has established that the BCIS has acceptable validity and reliability in both nonpsychiatric and psychotic samples. Consistent with the multidimensional view of cognitive insight, the CFAs supported a two-factor structure underlying the BCIS in non-psychiatric and psychotic populations. Most of GFI statistics performed better with GFI, AGFI, TLI, and RMSEA. However, two of them (CFI, NNFI) are slightly lower than the cut off previously recommended [55,56] and could be considered as acceptable. In CFA, previous studies have used the ratio χ**^2 ^**/df as an index to assess the consistency of different models' factor structure. Having χ**^2 ^**/df < 2 means that the model fits well, but it is important to note that the larger the sample size, the bigger the χ**^2 ^**[64]. Because of the large sample size (n = 507) in the present study, it might not be appropriate to use χ**^2 ^**/df as the index to assess the fit of the model.

The present findings add to the previous studies [38] that have suggested that participants with psychosis have impaired SR when compared with healthy controls. A partial explanation for this may lie in the fact that deficits in metacognition are a stable feature of schizophrenia [22] and that such deficits may be key features of the relationship between schizophrenia and self-reflectivity [17-19,21,22]. Although the same spectrum of cognitive dysfunctions should also be expressed in nonpsychiatric individuals, the severity of cognitive dysfunction should increase towards the schizophrenia end of the continuum. In other words, the core clinical and subclinical features of schizophrenia, such as limitations in the individuals' metacognitive capacity to reflect upon their difficulty in thinking as well as to recognize and correct their errors, will be found across the dimension of cognitive insight [17,18,21,22].

Previous studies investigating the relationship between cognitive insight and depression have reported conflicting results. Several studies have replicated the finding of Beck et al. [24] that depression was not related to cognitive insight in patients with a psychotic diagnosis [30-32,34]. Three studies [35,37,65], however, found that better cognitive insight (composite index and SR) is related to worse depression in patients with schizophrenia or schizoaffective disorder when looking at a cross-section. Our results replicate earlier findings [35,37,65] concerning the association between cognitive insight and depression in patients with schizophrenia. The results suggest that schizophrenia patients with depression have better cognitive insight than those without comorbid depression. This finding could be due to the fact that as individuals begin to believe they are mentally ill, they are confronted with and demoralized by self-stigma [17,45,66-70], which is, in turn, related to poor self-esteem [17,45,67-70], depressed mood [17,68], and hopelessness [17,68-70]. In empirical studies of this hypothesis, previous research has noted that the association of insight with depression [68], low quality of life [68], negative self-esteem [68,69], hope [69], and social functioning [69] is moderated by self-stigma. A previous study also reported that having a greater level of cognitive insight was significantly associated with experiencing a high level of self-stigma [45]. The increased SR or cognitive insight in such individuals can be used to indicate a negative attitude to self or "bad me" distortions of thinking [71,72], given that individuals with depression are characterized by a variety of negative biases about the self or negative self appraisal [72,73]. It may be of interest for future research to examine whether certain kinds of psychosocial intervention might be beneficial in improving SR or cognitive insight, while decreasing rather than increasing depressive symptoms. Furthermore, the relationships between cognitive insight and depression could be studied longitudinally. Unexpectedly, the present study found that depression was not significantly correlated with the BCIS index or its subscales in nonpsychiatric individuals. It is unlikely that this was due to a floor effect or a limited range of scores on the BDI (see Table 1). Thus, it can be reasoned that the relationship of the SR dimension of cognitive insight to depression is not driven by a specific cognitive process such as self-reflectivity but rather is related to the experience of adopting the stigmatizing label and internalizing the stigma. As with unexpected and negative findings, future studies are required before any firm conclusions can be drawn.

The area under the ROC curve in the present study demonstrated that the Taiwanese BCIS satisfactorily differentiated patients with schizophrenia from the nonpsychiatric controls. In addition to being useful as an outcome measure for assessing responses to cognitive insight, the BCIS might be employed as a screening instrument to identify patients who are willing to examine their erroneous beliefs and accept the types of corrective feedback afforded by cognitive therapy [26]. Colis et al. (2006) also suggested that the BCIS might be used to screen for patients who exhibit low levels of cognitive insight and require more stabilization with psychotropic medications before engaging in psychotherapy [35]. Therefore, in the present study, our goal was to validate use of the BCIS to screen for cognitive insight in clinical settings where one may want to maximize the sensitivity of a test. It is important to note that the risks associated with the profound damage of psychotic relapses and the difficulties in treating psychotic disorders are high. In addition, the costs associated with further assessment of those patients with psychosis who appear to be unaware of their illness, their need for treatment, and the social consequences of psychotic disorder are relatively low. Thus, a screening measure of cognitive insight that would identify this "high-risk" population would be valuable, providing good sensitivity at the cost of some specificity. For this reason, we suggested a BCIS index cutoff value of 3 (sensitivity, 87%; specificity, 51%) for the detection of moderate or severe impairments in cognitive insight in a Taiwanese population. However, cutoff scores provided in this and similar studies largely depend on sample characteristics (in particular, sample sizes and types of psychiatric disorders or conditions considered in the studies) as well as on the procedures used during assessment, diagnosis, and data analysis. Given the number of differences across studies in these and other variables, it is difficult to compare the current results with previous evidence; cutoff scores provided in this study should therefore be treated with caution [74].

Several methodological limitations should be considered when interpreting these findings. Most importantly, the size of the psychotic group was relatively small. Larger patient sample sizes would give more precise parameter estimates for CFA. Necessary sample sizes for CFA are controversial and require further research [75]. The rule of thumb that samples of 100-200 represent a "medium" sample size is not absolute because model complexity must also be considered [76]. Clearly, a larger sample size would provide more power for statistical tests. Second, generalizability may be limited given the particular sociodemographic characteristics of the study sample, which mainly included urban Taiwanese. Third, we readily acknowledge that our research was exploratory and that our recruitment procedure could be improved. The outpatients who agreed to participate in the cognitive insight assessment may have had better relationships with the staff, may have more clearly perceived the beneficial effects of treatment, or may have had a higher cognitive insight level than those who did not agree to participate. Fourth, in patients diagnosed with schizophrenia, deficits of metacognitive capacities or neurocognitive impairments are most likely the strongest predictor of cognitive insight [17,18,21,22] and future functional adaptability [19-21,23,77-80]. Determining reliable baseline cognitive function, particularly at the onset of the first episode of psychosis, may improve the predictive ability of these measures. In our study, however, cognitive insight assessments for patients with multiple relapses were limited due to the manifestations of neuropsychological deficits in schizophrenia spectrum disorders. Finally, none of the psychotic outpatients who participated in our study were naïve to antipsychotics. Indeed, all of the psychotic patients took atypical antipsychotics, and none of the patients were drug-free at the time of assessment. Specifically, studies have shown that atypical antipsychotics improve some aspects of cognitive functioning [81,82]. Further research in this area would benefit from the investigation of the influence of medication effects and neuropsychological deficits on cognitive insight formation in psychosis.

## Conclusions

Cognitive insight regarding mental illness may determine how people seek help for mental health problems or if they even seek help at all. Cognitive insight also may determine their level of engagement with treatment and the outcome of their problems. The usefulness of the BCIS in nonclinical and clinical samples was replicated in a Taiwanese population. This model enables the comparisons of the present results with the results from the US and other countries in which the same factor structure of BCIS items has been applied. The theoretical model underlying the BCIS provides related information; therefore, longitudinal studies are also recommended to analyze the antecedent-consequence relationship between dimensions of the scale in an empirical manner.

## Competing interests

The authors declare that they have no competing interests.

## Authors' contributions

YCK wrote draft of the manuscript. YCK, TSW, and YPL conceptualized and designed the study. YCK, TSW, and CWL collected and analyzed the data. YPL supervised the study. YCK analyzed the data further and wrote the final manuscript. YPL helped to draft and revised the manuscript. All authors read and approved the paper.

## Pre-publication history

The pre-publication history for this paper can be accessed here:

http://www.biomedcentral.com/1471-244X/11/170/prepub

## References

[B1] Amador XF Strauss DH Yale SA Gorman JM Awareness of illness in schizophrenia Schizophr Bull 1991 17 113 132 2047782 10.1093/schbul/17.1.113

[B2] David AS Insight and psychosis Br J Psychiatry 1990 156 798 808 10.1192/bjp.156.6.798 2207510

[B3] Morgan KD David AS Amador XF, David AS Neuropsychological studies of insight in patients with psychotic disorders Insight and Psychosis 2004 2 Oxford University Press 177 196

[B4] De Hert MAF Simon V Vidovic D Franic T Wampers M Peuskens J van Winkel R Evaluation of the association between insight and symptoms in a large sample of patients with schizophrenia Eur Psychiatry 2009 24 507 512 10.1016/j.eurpsy.2009.04.004 19540728

[B5] Lysaker PH Roe D Yanos PT Toward understanding the insight paradox: Internalized stigma moderates the association between insight and social functioning, hope, and self-esteem among people with schizophrenia spectrum disorders Schizophr Bull 2007 33 1 192 199 16894025 10.1093/schbul/sbl016 PMC2012366

[B6] Cooke MA Peters ER Kuipers E Kumari V Disease, deficit or denial? Models of poor insight in psychosis Acta Psychiatr Scand 2005 112 4 17 10.1111/j.1600-0447.2005.00537.x 15952940

[B7] Lysaker PH Lancaster RS Davis LW Clements CA Patterns of neurocognitive deficits and unawareness of illness in schizophrenia J Nerv Ment Dis 2003 191 38 44 10.1097/00005053-200301000-00007 12544598

[B8] Amador XF Flaum M Andreasen NC Strauss DH Yale SA Clark SC Gorman JM Awareness of illness in schizophrenia and schizoaffective and mood disorders Arch Gen Psychiatry 1994 51 826 836 7944872 10.1001/archpsyc.1994.03950100074007

[B9] Aleman A Agrawal N Morgan KD David AS Insight in psychosis and neuropsychological function: meta-analysis Br J Psychiatry 2006 189 204 212 10.1192/bjp.189.3.204 16946354

[B10] Flashman LA McAllister TW Andreasen NC Saykin AJ Smaller brain size associated with unawareness of ilness in patients with schizophrenia Am J Psychiatry 2000 157 1167 1169 10.1176/appi.ajp.157.7.1167 10873930

[B11] Flashman LA McAllister TW Johnson SC Rick JH Green RL Saykin AJ Specific frontal lobe subregions correlated with unawareness of illness in Schizophrenia: a preliminary study Journal of Neuropsychiatry & Clinical Neuroscience 2001 13 255 277 10.1176/appi.neuropsych.13.2.255 11449033

[B12] Sapara A Cooke MA Fannon D Francis A Buchanan RW Anilkumar APP Barkataki I Aasen I Kuipers E Kumari V Prefrontal cortex and insight in schizophrenia: a volumetric MRI study Schizophr Res 2007 89 22 34 10.1016/j.schres.2006.09.016 17097853

[B13] Amador XF Gorman JM Psychopathological domains and insight in schizophrenia Psychiatr Clin North Am 1998 21 27 42 10.1016/S0193-953X(05)70359-2 9551489

[B14] David AS "To see ourselves as others see us" Aubery Lewis's insight. Br J Psychiatry 1999 175 210 216 10.1192/bjp.175.3.210 10645320

[B15] Drake RJ Lewis SW Insight and neurocognition in schizophrenia Schizophr Res 2003 62 165 173 10.1016/S0920-9964(02)00382-1 12765757

[B16] Bora E Schitoglu G Asiler M Atabay I Veznedaroglu B Theory of mind and unawareness of illness in schizophrenia: is poor insight a mentalizing deficit? Eur Arch Psychiatry Clin 2007 257 2 104 111 10.1007/s00406-006-0681-3 17171312

[B17] Lysaker PH Buck KD Slvatore G Popolo R Dimaggio G Lack of awareness of illness in schizophrenia: conceptualization, correlates and treatment approaches Expert Rev Neurother 2009 9 7 1035 1043 10.1586/ern.09.55 19589052

[B18] Lysaker PH Dimaggio G Buck KD Callaway SS Salvatore G Carcione A Nicolo G Stanghellini G Poor insight in schizophrenia: links between different forms of metacognition with awareness of symptoms, treatment need, and consequences of illness Compr Psychiatry 2011 52 253 260 10.1016/j.comppsych.2010.07.007 21497218

[B19] Semerari A Carcione A Dimaggio G Falcone M Nicolo G Procacci M Alleva G How to evaluate matacognitive function in psychotherapy? The Metacognition Assessment Scale and its application Clin Psychol Psychother 2003 10 238 261 10.1002/cpp.362

[B20] Lysaker PH Warman DM Dimaggio G Procacci M LaRocco VA Clark LK Dike CA Nicolo G Metacognition in schizophrenia: Associations with multiple assessments of executive function J Nerv Ment Dis 2008 196 5 384 389 10.1097/NMD.0b013e3181710916 18477880

[B21] Lysaker PH Dimaggio G Carcione A Procacci M Buck KD Davis LW Nicolo G Metacognition and schizophrenia: The capacity for self-reflectivity as a predictor for prospective assessments of work performance over six months Schizophr Res 2010 122 124 130 10.1016/j.schres.2009.04.024 19457645

[B22] Lysaker PH Olesek KL Warman DM Martin JM Salzman AK Nicolo G Salvatore G Dimaggio G Metacognition in schizophrenia: Correlates and stability of deficits in theory of mind and self-reflectivity Psychiatry Res 2010 in press 10.1016/j.psychres.2010.07.016 20696482

[B23] Lysaker PH McCormick BP Snethen G Buck KD Hamm JA Megan G Nicolo G Dimaggio G Metacognition and social function in schizophrenia: Associations of mastery with functional skills competence Schizophr Res 2011 131 241 218 10.1016/j.schres.2011.06.011 21745724

[B24] Beck AT Baruch E Balter JM Steer AS Warman DM A new instrument for measuring insight: The Beck Cognitive Insight Scale Schizophr Res 2004 68 319 329 10.1016/S0920-9964(03)00189-0 15099613

[B25] Beck AT Warman DM Amador XF, David AS Cognitive insight: theory and assessment Insight and Psychosis: Awareness of Illness in Schizophrenia and Related Disorders 2004 2 Oxford, UK: Oxford University Press 3 30

[B26] Granholm E McQuaid JR McClure FS Auslander LA Perivoliotis D Pedrelli P Patterson T Jeste DV A randomized, controlled trial of cognitive behavioural social skills training for middle-aged and older outpatients with chronic schizophrenia Am J Psychiatry 2005 162 520 529 10.1176/appi.ajp.162.3.520 15741469

[B27] Cooke MA Peters ER Fannon D Aasen I Kuipers E Kumari V Cognitive insight in psychosis: The relationship between self-certainty and self-reflection dimensions and neuropsychological measures Psychiatry Res 2010 178 284 289 10.1016/j.psychres.2009.05.009 20483170 PMC3184477

[B28] Bora E Erkan A Kayahan B Veznedaroglu B Cognitive insight and acute psychosis in schizophrenia Psychiatry Clin Neurosci 2007 61 634 639 10.1111/j.1440-1819.2007.01731.x 18081624

[B29] Lepage M Buchy L Bodnar M Bertrand M-C Joober R Malla A Cognitive insight and verbal memory in first episode psychosis Eur Psychiatry 2008 23 368 374 10.1016/j.eurpsy.2008.02.003 18374546

[B30] Tranulis C Lepage M Malla A Insight in first episode psychosis: who is measuring what? Early Interv Psychiatry 2008 2 34 41 10.1111/j.1751-7893.2007.00054.x 21352129

[B31] Favrod J Zimmermann G Raffard S Pomini V Yasser Khazaal Y The Beck Cognitive Insight Scale in outpatients with psychotic disorders: further evidence from a French-Speaking sample Can J Psychiatry 2008 53 11 783 787 19087473 10.1177/070674370805301111

[B32] Engh JA Friss S Birkenaes AB Measuring cognitive insight in schizophrenia and bipolar disorder: A comparative study BMC Psychiatry 2007 7 71 77 10.1186/1471-244X-7-71 18072961 PMC2222246

[B33] Uchida T Matsumoto K Kikuchi A Miyakoshi T Ito F Ueno T Matsuoka H Psychometric properties of the Japanese version of the Beck Cognitive Insight Scale: relation of cognitive insight to clinical insight Psychiatry Clin Neurosci 2009 63 291 297 10.1111/j.1440-1819.2009.01946.x 19566759

[B34] Pedrelli P McQuaid JR Granholm E Patterson TL McClure F Beck AT Jeste DV Measuring cognitive insight in middle-aged and older patients with psychotic disorders Schizophr Res 2004 71 297 305 10.1016/j.schres.2004.02.019 15474900

[B35] Colis MJ Steer RA Beck AT Cognitive insight in inpatients with psychotic, bipolar, and major depressive disorders J Psychopathol Behav Assess 2006 28 242 249 10.1007/s10862-005-9012-7

[B36] Warman DM Martin JM Cognitive insight and delusion proneness: An investigation using the Beck Cognitive Insight Scale Schizophr Res 2006 84 297 304 10.1016/j.schres.2006.02.004 16545944

[B37] Warman DM Lysaker PH Martin JM Cognitive insight and psychotic disorder: The impact of active delusions Schizophr Res 2007 90 325 333 10.1016/j.schres.2006.09.011 17092694

[B38] Martin JM Warman DM Lysaker PH Cognitive insight in non-psychiatric individuals and individuals with psychosis: an examination using the Beck Cognitive Insight Scale Schizophr Res 2010 121 39 45 10.1016/j.schres.2010.03.028 20399611

[B39] Moore O Cassidy E Carr A O'Callaghan E Unawareness of illness and its relationship with depression and self-deception in schizophrenia Eur Psychiatry 1999 14 264 269 10.1016/S0924-9338(99)00172-8 10572356

[B40] Mohamed S Rosenheck R McEvoy J Swartz M Stroup S Lieberman JA Cross-sectional and longitudinal relationships between insight and attitudes toward medication and clinical outcomes in schizophrenia Schizophr Bull 2009 35 2 336 346 10.1093/schbul/sbn067 18586692 PMC2659303

[B41] Kao YC Liu YP The clinical applicability of the Self-Appraisal of Illness Questionnaire (SAIQ) to chronic schizophrenic patients in Taiwan Psychiatr Q 2010 81 3 215 225 10.1007/s11126-010-9131-5 20364323

[B42] Engh J Friis S Birkenaes AB Jonsdottir H Klungsoyr O Ringen PA Delusions are associated with poor cognitive insight in schizophrenia Schizophr Bull 2010 36 4 830 835 10.1093/schbul/sbn193 19176474 PMC2894586

[B43] Carlson J Ochoa S Haro JM Escartín G Ahuir M Gutierrez-Zotes A Salamero M Valero J Cañizares S Bernardo M Cañete J Gallo P Adaptation and validation of the quality of life scale: satisfaction with Life Domains Scale by Baker and Intaglita Compr Psychiatry 2009 50 76 80 10.1016/j.comppsych.2008.05.008 19059518

[B44] Kim HJ Jhin HK Chung EK Chang D Lee J Cross-cultural validation of the Beck Cognitive Insight Scale in Korean Psychiatry Invest 2007 4 109 115

[B45] Mak WWS Wu CFM Cognitive insight and causal attribution in the development of self-stigma among individuals with schizophrenia Psychiatry Serv 2006 57 1800 1802 10.1176/appi.ps.57.12.1800 17158498

[B46] Kao YC Liu YP The Beck Cognitive Insight Scale (BCIS): translation and validation of the Taiwanese version BMC Psychiatry 2006 10 27 10.1186/1471-244X-10-27 PMC2873466 20377914

[B47] Krzanowski WJ Hand DJ ROC curves for continuous Data 2009 Chapman & Hall/CRC, Boca Raton, FL

[B48] American Psychiatric Association Diagnostic and Statistical Manual of Mental Disorders 2000 4 Washington (DC), American Psychiatric Press

[B49] Cockrell JR Folstein MF Mini-Mental State Examination (MMSE) Psychopharmacol Bull 1988 24 689 692 3249771

[B50] Beck AT Steer RA Brown GK Beck Depression Inventory Manual 1996 2 Psychological Corporation, Harcourt, Brace, San Antonio, TX

[B51] Nunnally JC Bernstien IH Psychometric Theory 1994 3 New York, MacGraw-Hill

[B52] Anderson JC Gerbing DW The effect of sampling error on convergence, improper solution, and goodness-of-fit indices for maximum likelihood confirmatory factor analysis Psychometrika 1984 49 155 173 10.1007/BF02294170

[B53] Bentler PM Chou CP Practical issues in structural modeling Sociol Methods Res 1987 16 78 117 10.1177/0049124187016001004

[B54] Garson GD Structural equation modeling. Statnotes: topics in multivariate analysis [Internet] 2007 Raleigh (NC): North Carolina State University

[B55] Hoyle RH Panter AT Hoyle RH Writing about structural equation modeling Structural equation modeling: Concepts, issues, and applications 1995 Thousand Oaks, Park, CA, Sage

[B56] Petersens S Hagglof B Stenlund H Bergstrom E Psychometric properties of the Swedish PedsQL, Pediatirc Quality of Life Inventory 4.0 generic core scales Acta Paediatrica 2009 98 1504 1512 10.1111/j.1651-2227.2009.01360.x 19570060

[B57] Youden WJ Index for rating diagnostic tests Cancer 1950 3 32 35 10.1002/1097-0142(1950)3:1<32::AID-CNCR2820030106>3.0.CO;2-3 15405679

[B58] Faraggi D The effect of random measurement error on receiver operating characteristic (ROC) curves Stat Med 2000 19 61 70 10.1002/(SICI)1097-0258(20000115)19:1<61::AID-SIM297>3.0.CO;2-A 10623913

[B59] Reiser B Measuring the effectiveness of diagnostic markers in the presence of measurement error through the use of ROC curves Stat Med 2000 19 2115 2129 10.1002/1097-0258(20000830)19:16<2115::AID-SIM529>3.0.CO;2-M 10931515

[B60] Hanley JA McNeil BJ The meaning and use of the area under a receiver operating characteristic (ROC) curve Radiology 1982 143 29 36 7063747 10.1148/radiology.143.1.7063747

[B61] Hosmer DW Lemeshow S Applied logistic Regression 1989 2 Willey, New York

[B62] Cortina JM What is coefficient alpha? An examination of theory and applications J Appl Psychol 1993 78 98 104

[B63] Holden RR Fekken GC Cotton DH Assessing psychopathology using structured test-item response latencies Psychol Assess 1991 3 111 118

[B64] Wang Q Vassos E Deng W Ma X Hu X Murray RM Collier DA Li T Factor structures of the neurocognitive assessments and familiar analysis in first-episode schizophrenia patients, their relatives and controls Aust N Z J Psychiatry 2010 44 109 119 10.3109/00048670903270381 20113299

[B65] Ugurlu GK Ekinci O Albayrak Y Arslan M Caykoylu A The relationship between cognitive insight, clinical insight, and depression in patients with schizophrenia Compr Psychiatry 2011 in press 10.1016/j.comppsych.2011.02.010 21489416

[B66] Corrigan PW How clinical diagnosis might exacerbate the stigma of mental illness Social Work 2007 52 31 39 17388081 10.1093/sw/52.1.31

[B67] Watson AC Corrigan P Larson JE Sells M Self-stigma in people with mental illness Schizophr Bull 2007 33 6 1312 1318 17255118 10.1093/schbul/sbl076 PMC2779887

[B68] Staring ABP Van der Gaag M Van den Berge M Duivenvoorden HJ Mulder CL Stigma moderates the associations of insight with depressed mood, low self-esteem, and low quality of life in patients with schizophrenia Schizophr Res 2009 115 363 369 10.1016/j.schres.2009.06.015 19616414

[B69] Lysaker PH Roe D Yanos PT Toward understanding the insight paradox: Internalized stigma moderates the association between insight and social functioning, hope, and self-esteem among people with schizophrenia spectrum disorders Schizophr Bull 2007 33 1 192 199 16894025 10.1093/schbul/sbl016 PMC2012366

[B70] Lysaker PH Tsai J Yanos P Roe D Association of multiple domains of self-esteem with four dimensions of stigma in schizophrenia Schizophr Res 2008 98 194 200 18029145 10.1016/j.schres.2007.09.035 PMC3208262

[B71] Trower P Chadwick PDJ Pathways to defense to defense of the self: a theory of two types of paranoia Clin Psychol Sci Prac 1995 2 263 278 10.1111/j.1468-2850.1995.tb00044.x

[B72] Riggs SE Grant PM Perivoliotis D Beck AT Assessment of Cognitive Insight: A Qualitative Review Schizophr Bull 2010 in press 10.1093/schbul/sbq085 PMC3283158 20693342

[B73] Beck AT Alford BA Depression: Causes and Treatment 2009 2 Philadelphia, PA: University of Pennsylvania Press

[B74] Rivas T Bersabe R Jimenez M Berrocal C The eating attitudes test (EAT-26): reliability and validity in Spanish female samples Span J Psychol 2010 13 2 1044 1056 20977051 10.1017/s1138741600002687

[B75] Jackson DL Revisiting sample size and number and number of parameter estimate: some support for the N: q hypotheses Structural Equation Modeling 2003 10 128 141 10.1207/S15328007SEM1001_6

[B76] Kline RB Principles and practice of Structural Equation Modeling 2005 2 Guilford Press, New York

[B77] Green MF Kern RS Braff DF Mintz J Neurocognitive deficits and functional outcome in schizophrenia: Are we measuring the right stuff? Schizophr Bull 2002 26 119 136 10.1093/oxfordjournals.schbul.a033430 10755673

[B78] Davis LW Eicher AC Lysaker PH Metacognition as a predictor of therapeutic alliance over 26 weeks of psychotherapy Schizophr Res 2011 129 85 90 10.1016/j.schres.2011.02.026 21458241

[B79] Lysaker PH Shea AM Buck KD Dimaggio G Nicolo G Procacci M Salvatore G Rand KL Metacognition as a mediator of the effects of impairments in neurocognition on social function in schizophrenia spectrum disorders Acta Psychiatr Scand 2010 122 405 413 10.1111/j.1600-0447.2010.01554.x 20346074

[B80] Orfei MD Spoletini I Banfi G Caltagirone C Spalletta G Neuropsychological correlates of cognitive insight in schizophrenia Psychiatry Res 2010 178 51 56 10.1016/j.psychres.2009.05.013 20452049

[B81] Harvey PD Keefe RSE Studies of cognitive change in patients with schizophrenia following novel antipsychotic treatment Am J Psychiatry 2001 158 176 184 10.1176/appi.ajp.158.2.176 11156796

[B82] Meltzer HY McGurk SR The effects of clozapine, risperidone, and olanzapine on cognitive function in schizophrenia Schizophr Bull 1999 25 233 255 10416729 10.1093/oxfordjournals.schbul.a033376

